# Structural Characteristics and Immunomodulatory Effects of a Long-Chain Polysaccharide From *Laminaria japonica*

**DOI:** 10.3389/fnut.2022.762595

**Published:** 2022-03-28

**Authors:** Jiamei Cui, Yunpeng Wang, Eunyoung Kim, Chongyu Zhang, Guiguo Zhang, Yunkyoung Lee

**Affiliations:** ^1^Department of Food Science and Nutrition, Jeju National University, Jeju, South Korea; ^2^Shandong Provincial Key Laboratory of Animal Biotechnology and Disease Prevention, Department of Animal Nutrition, Shandong Agricultural University, Taian City, China; ^3^Interdisciplinary Graduate Program in Advanced Convergence Technology and Science, Jeju National University, Jeju, South Korea

**Keywords:** *Laminaria japonica*, polysaccharide, structural characteristics, bioactivity, 3D structure analysis

## Abstract

Polysaccharides derived from *Laminaria japonica* (LJPS) have shown a variety of beneficial effects on improving human health; however, the structural features and bioactivities of long-chain LJPS remain unclear. This study aimed to investigate the structural characteristics and bioactivities of a novel long-chain LJPS. Results showed that the LJPS was composed of Fuc, Rha, Ara, Gal, Glc, Xyl, Man, Fru, Rib, GalA, GluA, GlcA, and ManA, with a molar ratio of 35.71:1.48:0.28:13.16:0.55:2.97:6.92:0.58:0.41:0.14:3.16:15.84:18.79. Of these, Fuc, Gal, Man, GlcA, and ManA were the predominant components with an accumulated proportion of 93.6%. The LJPS was found to consist of seven types of the monomer residues, and the main interchain glycosidic linkages were **β** -D-(1 → 2), **α** -D-(1 → 3), (1 → 4), and (1 → 6), and the molecular mass was 5.79 × 10^4^ g/mol. Regarding the molecular conformation, LJPS was a multi-branched, long-chain macromolecule, and appeared in a denser crosslinking network with highly branched and helix domains in the terms of morphology. Additionally, the LJPS had no toxicity to mouse macrophage cells and exhibited biphasic immuno-modulating capacity. The present findings suggested that the long-chain LJPS might be an attractive candidate as an immunopotentiating and anti-inflammatory functional food, and this study also provides a feasible approach to decipher the structural characteristics and spatial conformations of plant-derived polysaccharides.

## Introduction

*Laminaria japonica* (LJ), rich in soluble polysaccharide (PSs), has been widely utilized as not only an important dietary component but also a traditional medicine in many Asian countries (1). This high dietary seaweed consumption is associated with a decreased risk of diabetes mellitus and low metabolic syndrome prevalence in certain populations (2, 3). Emerging data have demonstrated that these special nutritional and medicinal biofunctions of LJ are tightly related to the polysaccharides contained in this plant.

Additionally, the PSs extracted from LJ exhibit a variety of health-beneficial biofunctions, such as anti-oxidation (4, 5), anti-inflammatory (6, 7), lipid-lowering (8, 9), anti-diabetes (10), and anti-obesity effects (11). Of note, the discrepant bioactivities of LJ-derived PS (LJPS) might occur owing to different isolation procedures or sources (7, 12, 13) and more likely due to the differentiated molecular structure of the LJPS. Some studies have indeed suggested that the biofunctions of plant-derived PSs are defined by their unique structural characteristics (14–16).

Regarding the molecular structure of LJPS, most current research has focused on the analyses of monosaccharide composition, molecular weight, glycosidic linkages, and substituted groups *via* high-performance liquid chromatography (HPLC), near-infrared spectroscopy, gel penetration chromatography, and other methods. Moreover, with respect to the conventional indicators of molecular structure, recent studies have addressed the pivotal role of PS chain length (degree of polymerization, DP) and spatial conformation in their bioactivities (11, 17, 18). Similarly, many studies on inulin and fructan have shown that DP value is one of the most crucial factors affecting their biological functions (11, 19). For example, Li et al. (19) observed that a short-chain inulin (DP = 4–5) increased blood glucose peaks, whereas a long-chain inulin (DP = 23–25) had superior effects on glucose homeostasis and total cholesterol control compared with those of the short-chain inulin. In addition, the long-chain inulin was able to preferentially promote the proliferation of intestinal *Bacteroides* (19), resulting in the production of a series of enzymes that degrades complex carbohydrates into oligosaccharides and monosaccharides (20, 21). Furthermore, the long-chain inulin (DP = 3–60) results in the production of more short-chain fatty acids (SCFAs) in the gut and *in vitro* fermentation compared with those with oligofructose (DP = 2–20) or short-chain inulin (22). In addition, the DP affects the prebiotic effects of fructans, and high polymerization facilitates the increased alpha-diversity and acidification of the gut microbial community (17). Thus, it was reported that plant-derived PSs with different polymerization properties exert different bioeffects (12, 23). Therefore, PSs with long chains or a high DP might have superior effects on promoting beneficial intestinal microbiota and other biological activities. Similarly, Gao et al. (24) documented that LJPSs with a highly branched structure and denser interconnected macromolecule network indeed have greater bile acid-binding capacity.

Notwithstanding, how the structural characteristics and bioactivities of long-chain LJPSs are interconnected remains largely unknown. A better understanding of the association between structure and biological properties would provide a clue to determine the potential bioactivity of previously known and newly discovered LJPSs based on their structural characterization (25). However, most previous studies on LJPS have focused on the analyses of the monosaccharide composition, molecular weight, glycosidic linkages, and other molecular characteristics of PSs. These isolated molecular features cannot provide the overall spatial shape of PS molecules.

Thus, we hypothesized that long-chain LJPSs would have a unique molecular structure that affects their bioactivities. The objectives of this study were (1) to decipher the structural characteristics and integrate those structural parameters to determine the spatial three-dimensional (3-D) molecular conformation of long-chain LJPSs (DP > 20) and (2) to investigate the immunomodulatory effects of long-chain LJPSs using the RAW 264.7 mouse macrophage cell line. This would provide a better understanding of the structural characteristics and bioactivities of long-chain LJPSs.

## Materials and Methods

### Extraction and Purification of Long-Chain Polysaccharides From *Laminaria japonica*

*Laminaria japonica* was purchased from a traditional market in Jeju, South Korea. The PSs from LJ were extracted and purified following the methods described by Zhang et al. (26) with some modifications to enhance the isolation efficiency. Briefly, the fresh LJ was washed with tap water to remove cohesive salt and chopped into 2–3 cm-long pieces and oven-dried at 65°C to a constant weight. The prepared sample was placed into a big glass beaker and mixed with double-distilled water (d-H_2_O) in the ratio of 1:30 (*v*:*v*, LJ:d-H_2_O), boiled for 4 h, and subsequently filtered through the two layers of nylon mesh (0.2 mm mesh). The filtrate was continuously heated and kept slightly boiling to concentrate the filtrate to one-fourth volume from the initial volume. After cooling, the remaining filtrate was mixed with trichloroacetic acid (TCA) solution (5% concentrate) at a proportion of 1:2 (*v*:*v*, filter liquid:TCA) and maintained for 2 h statically to remove protein from the liquid. After the protein was precipitated completely, the liquid fraction was centrifuged at 3,000 × *g* for 10 min to collect the supernatant liquid, and the sediment was discarded; then, the supernatant was transferred to another container and a 4-fold volume of absolute ethyl alcohol (*v*:*v*) was added. The mixture was kept at 4°C for 12 h and then centrifuged at 3,000 × *g* for 10 min to separate the precipitated crude PS. The fractionated crude PS was subsequently re-dissolved in d-H_2_O and dialyzed two times using an ultrafiltration membrane (molar mass > 3,500 D Mw, DP > 20, Beijing Solarbio Science and Technology Co., Ltd., Beijing, China) against d-H_2_O (10 times the sample volume) at 4°C for 48 h, changing the d-H_2_O every 12 h. The dialyzed liquid was collected and lyophilized to a constant weight using a vacuum dryer (Biosafer-10A, Biosafer, Nanjing, China), which was considered the crude PSs from LJ (LJPSs). Further purification was performed using anion-exchange column chromatography. Then, 3 g of crude PSs was dissolved in 100 ml of d-H_2_O and then applied to a DEAE-52 anion-exchange column (5.0 cm × 100.0 cm). A stepwise gradient NaCl aqueous solution (0, 0.2, and 0.4 mol/L) was used for elution at a flow rate of 0.5 ml/min. Three fractions were collected separately and the concentration of polysaccharide in elution was detected with the phenol–sulfuric acid method (24, 27). The absorbance peak of 0.4 mol/L was much larger than the others, therefore, the fraction eluted with 0.4 mol/L NaCl was further purified using gel-permeation chromatography with a Sephacryl S-500, yielding one homogeneous fraction LJPSs (28, 29). The column was washed with d-H_2_O at a rate of 0.2 ml/min, and eluted samples were freeze-dried. The sulfate radicals were analyzed with barium chloride gelatin analysis following the method described by Li et al. (27), and the total phenol contents were determined by the liquid chromatography electrospray ionization mass spectrometric (LC-ESI-MS) method.

### Determination of Monosaccharide Compositions of LJ-Derived PS

The monosaccharide compositions of LJPS were identified by ion chromatography (IC). The detailed operation procedure was as follows: the absorption curve was established by measuring the absorbance of monosaccharide standards. In total, 13 standard substrates were used in the present study, such as fucose (Fuc), rhamnose (Rha), arabinose (Ara), galactose (Gal), glucose (Glc), xylose (Xyl), mannose (Man), fructose (Fru), ribose (Rib), galacturonic acid (GalA), glucuronic acid (GlcA), guluronic acid (GulA), and mannuronic acid (ManA). The standard sample of each monosaccharide was accurately weighed as 10.00 mg and dissolved in 10 ml of ultrapure water in 10 ml volumetric flasks to obtain 1, 5, 8, 16, 30, 40, 50, and 60 μg/ml standard solutions. Each standard solution was analyzed by IC (Thermo Fisher Scientific, ICS5000 type, Waltham, MA, United States) and a diode-array detector (Young-Lin Co., Changsha, China). The flow phases were NaOH (200 mM) or NaAC (200 mM) and the flow rate was 1.0 ml/min. The sample injection volume was 100 μl. The standard curve for each monosaccharide was fitted according to the retention time and absorption peak. A 10 mg (± 0.005) LJPS sample was accurately weighted into a glass tube, to which 1 ml of 2.5 mol/L trifluoroacetic acid was added for 90 min acidolysis at 110°C. The acidolysis mixture was dried in a vacuum rotator, and 5 ml of sterile water was added to fully dissolve the residue. The resulting solution was centrifuged at 12,000 × *g* for 10 min, and the supernatant was collected to determine the components by IC following the procedure mentioned previously herein. The retention time was matched with the monosaccharide standard curve to determine the monosaccharide composition of the LJPS sample. The molar amount of each component was calculated from the peak area of each derivative.

### Determination of Molecular Weight of LJ-Derived PS

In this study, the molecular weight of LJPS was evaluated using the following parameters (26, 30): the number-average molecular weight (Mn), expressed as Mn = Σ(niMi)/Σni; the weight-average molecular weight (Mw), defined as Mw = Σ(niMi^2^)/Σ(niMi); and the *Z*-average molecular weight (Mz), calculated as Mz = Σ(niMi^3^)/(ΣniMi^2^) (26, 30). The polydispersity coefficient presents the range of the molecular mass distribution and can be calculated by dividing Mw by Mn or dividing M_*Z*_ by Mn. The purified LJPS samples were accurately weighed as 5.00 mg and dissolved in 1 ml of 90% dimethyl sulfoxide (DMSO) in a glass tube for an overnight incubation in a 100°C water bath, to which 3 ml of absolute ethanol (analyzed as pure, Sinopharm, Beijing, China) was added and then mixed vigorously. The mixture was centrifuged at 1,000 × *g* for 5 min, and the supernatant was removed. Then, the residual sediment was rinsed two times with anhydrous ethanol, and 3 ml of 0.1 M NaNO_3_ (containing 0.02% NaN_3_, Sigma Aldrich, Shanghai, China) was added for a 20 min incubation at 121°C. The mixture was centrifuged at 12,000 × *g* for 10 min, and the supernatant was collected and assessed by the gel permeation chromatography-refractive index-multiangle laser light scattering (GPC-RI-MALS; Dawn Heleos II, Wyatt Technology, Santa Barbara, CA, United States) method. The eluent was a 0.1 M NaNO_3_ and 0.02% NaN_3_ mixture (1:1, *v*/*v*; HPLC, Sigma-Aldrich, Shanghai, China). The flow rate was maintained at 0.4 ml/min, and the temperature of the column was maintained at 60°C. The eluent was monitored with a refractive index (RI) detector (Agilent 1260, Guangzhou, China); the analytic column included an Ohpak SB-805 HQ, Ohpak SB-804 HQ, and Ohpak SB-803 HQ (Shodex, Asahipak, Tokyo, Japan). The volume of the manually injected sample was 300 μl. Mass spectra were viewed and analyzed with ASTRA6.1 software (Wyatt Technology Corporation, Santa Barbara, CA, United States).

### Glycosidic Linkages and Conformation of LJ-Derived PS

The methylation analysis of PS was performed according to a previous method (31, 32) with minor modifications. In brief, 3 mg LJPS powder was accurately weighed and dissolved in 1 ml d-H_2_O, and added into 200 μl 2-morpholine ethane sulfonic acid (0.2 M) and 200 μl carbodiimide (500 mg/ml) solution, reacting for 2 h. The reaction mixture was subsequently added with 1 ml imidazole (4 mol/L) and divided into two equal parts, then, one of the part was mixed and reacted with 1 ml NaBH_4_ (30 mg/ml) and the other was mixed and reacted with 1 ml NaBD_4_ (30 mg/ml) for another 3 h until the termination of reaction by adding 200 μl acetic acid. The two reacted solutions were then dialyzed for 48 h followed by freeze-drying. The dried samples were individually dissolved in 500 μl anhydrous DMSO and reacted with 50 μl DMSO/NaOH (120 mg/ml) solution for 30 min. Then, 10 ml of CH_3_I solution was added to the reaction and incubated another 10 min followed by combining with 1 ml H_2_O and 500 μl of dichloromethane (DCM) solution to obtain DCM phase. The dried samples were then dissolved in 100 μl trifluoroacetic acid (2 M), reacted at 121°C for 90 min followed by evaporation to dryness at 30°C. The sample was dissolved in 50 μl of ammonia H_2_O (2 M), mixed and reacted with 50 μl of 1 M NaBD_4_ for 2.5 h. Further 20 μl of acetic acid was added to the mixture to end the reaction, followed with washing it two times with 250 μl of methanol, and dried under nitrogen. The obtained powder was mixed and reacted with 250 μl of acetic anhydride at 100°C for 2.5 h followed by mixed and incubated with 1 ml of H_2_O for another 10 min. Finally, the sample was mixed with 500 μl of DCM, and centrifuged to discard aqueous phase, and the bottom DCM phase were tested on the machine. A 6890A-5975C gas chromatography mass spectrometry (GC-MS) system (Agilent Technologies Inc., CA, United States) equipped with a BPX70 capillary column (30 m × 0.25 mm, 0.25 μm) was used to analyze the glycosidic linkages. The gas chromatographic conditions were as follows: BPX70 chromatographic column; high purity helium gas was used as the carrier gas at a flow rate of 1.0 ml/min; the injection volume was 1 μl, and the split ratio was 10:1; the initial column temperature was 140°C, retaining this for 2 min, and procedurally increasing it to 230°C at 3°C/min, with subsequent holding for 5 min; the injection temperature was 230°C; the ion source of the mass spectrometer was set at 230°C, and the four stage bar temperature was 150°C. The resulting peaks of alditol acetates were identified based on their MS fragmentation patterns and the relative retention time in the GC spectrum. The molar ratios of individual linkage residues were estimated as the ratios of peak areas.

### Determination of LJ-Derived PS Microstructures

The surface topography and microstructure of long-chain LJPS can be imaged by scanning electron microscopy (SEM). When the PS was dissolved in water or other solvents, its macromolecules with its three-dimensional structure would stretch into the unfolded chain or net status owing to the liquid tension, which can be inspected by transmission electron microscopy (TEM). The brief procedure was as follows: dried LJPS powder was scattered on a metal stub, sputtered with gold, and then examined by SEM with the model JEOL (JSM-IT100, Tokyo, Japan). The images were taken at different magnifications (× 200 and × 2,500). For TEM inspection, a well-dispersed PS solution was first prepared using sodium dodecyl sulfate (SDS, 5 μg/ml) to a final concentration of 5 μg/ml. The SDS aqueous solution was applied to dissolve LJPS as the SDS solution could reduce molecular aggregation. A droplet of LJPS solution (5 μl) was deposited on a carbon film specimen (200 mesh, Beijing Zhongjingkeyi Technology, Beijing, China) and dried at room temperature (25 ± 2°C). The specimen was examined using TEM (Tecnai G2 Spirit BIOTWIN, FEI, Hillsboro, OR, United States) at an accelerating voltage of 100 kV to observe the microstructures of LJPS.

### Spectroscopic Analysis

The infrared spectrum of LJPS was measured on a Fourier transforms infrared (FT-IR) spectrometer (FT-IR 650, Tianjin Gangdong Sci. &Tech. Co., Ltd., China) in the wavenumber range of 400-4,000 cm^–1^ by pressing LJPS samples (2 mg) and KBr (200 mg) into a pellet. One- and two-dimensional nuclear magnetic resonance (NMR) analyses of LJPS were carried out on an Avance Bruker III HD 600 MHz NMR spectrometer equipped with a 5 mm TCl CryoProbe at 25°C using D_2_O as the solvent at a final sample concentration of 40 mg/ml. The molecular structure of LJPS was determined *via* the analysis of one- and two-dimensional NMR spectra, such as the ^1^H-NMR, ^13^C-NMR, heteronuclear single quantum coherence (C, H-HSQC), single-bond proton–proton correlation spectroscopy (H, H-COSY), heteronuclear multiple bond coherence (H, C-HMBC), and nuclear Overhauser effect spectroscopy (H, H-NOESY).

### Bioactivities of LJ-Derived PS

#### Cell Culture and Cellular Toxicity

The RAW 264.7 cells, which are murine macrophages, were purchased from ATCC (Manassas, VA, United States), and the cellular toxicity of LJPS was determined by 3-(4,5-dimethyl thiazol-2-yl)-2,5-diphenyl tetrazolium bromide (MTT) assays as described previously (33). Cells were cultured in high-Glc Dulbecco’s modified Eagle’s medium supplemented with 10% fetal bovine serum and 1% P/S at 37°C in 5% CO_2_. For MTT assay, cells (0.25 × 10^6^ cells/well) were seeded with serum-starved media (containing 1% FBS) in a 24-well plate. On the following day, the cells were treated with the different doses of LJPS for 6 h with or without lipopolysaccharide (LPS; Sigma-Aldrich, St. Louis, MO, United States; 100 ng/mL) for another 18 h. After removing the treatment media, 100 μl of 2 mg/ml MTT solution was added, and cells were incubated at 37°C for another 3 h. The medium was removed, and 100 μl of DMSO was then added. The absorbance was measured at 540 nm with a microplate reader (Molecular devices, San Jose, CA, United States). All materials for cell culture were purchased from Gibco (BRL, Gaithersburg, MD, United States).

#### Measurement of Nitric Oxide and Proinflammatory Cytokines Induced by LJ-Derived PS in RAW 264.7 Cells

RAW 264.7 cells (1 × 10^6^ cells/well) were plated in a 6-well plate. On the following day, the cells were treated with LJPS for 6 h and then stimulated with LPS (100 ng/ml) for another 18 h. At the end of incubation, the supernatant was collected to measure nitric oxide (NO) production with the Griess reagent (Sigma-Aldrich, St. Louis, MO, United States) as described elsewhere (33). Briefly, the supernatant (50 μl) was mixed with 50 μl of the Griess reagent in a 96-well plate and incubated at room temperature for 15 min. The nitrite concentrations were measured using a standard curve prepared from the different concentrations of sodium nitrite. Absorbance was measured at 540 nm with a microplate reader. The relative NO production was calculated based on the LPS-stimulated group, which was considered 100%. Total RNA was extracted from the RAW 264.7 cell using TRIzol reagent (Invitrogen, Carlsbad, CA, United States). cDNA synthesis was performed using an ABI High Capacity cDNA Archive kits (Thermo Fisher Scientific, CA, United States) according to the manufacturer’s instructions. cDNA samples were diluted with RNase Free Water, and real-time PCR was performed with SYBR (Bio-Rad^®^, CA, United States), and specific targeting forward and reverse primers were as follows: tumor necrosis factor (*Tnf*)-α forward, GGCTGCCCCGACTACGT; *Tnf*-α reverse, ACTTTCTCCTGGTATGAGATAGCAAAT; interleukin (*Il*)-6 forward, CTGCAAGAGACTTCCATCCAGTT; and *Il-6* reverse, AGGGAAGGCCGTGGTTGT. Relative gene expression, which was normalized to the levels of ribosomal protein lateral stalk subunit P0 (*Rplp0, 36b4*), was determined by real-time PCR (CFX96™Real-TimePCR Detection System, Bio-Rad, Hercules, CA, United States). In addition, ELISA was performed to detect IL-6 and TNF-α (BD PharMingen, San Jose, CA, United States) to measure secreted protein levels in supernatant collected from RAW 264.7 cells according to the manufacturer’s instructions. Absorbance was measured at 450–570 nm with a microplate reader.

### Statistical Analysis

Data were analyzed by one-way analysis of variance (ANOVA). Tukey’s family error rate was used for a one-way multiple comparison (*p* < 0.05) with GraphPad Software, Prism 8.0.1 (San Diego, CA, United States). Values were expressed as the mean ± SEM. The 3d structure and spatial configurations of the LJPS molecule were speculated using the online analysis GLYCAM^1^ by integrating the monomer compositions, glycosidic linkages, and NMR information.

## Results

### Apparent Status and Monomer Compositions of LJ-Derived PS

**FIGURE 1:**
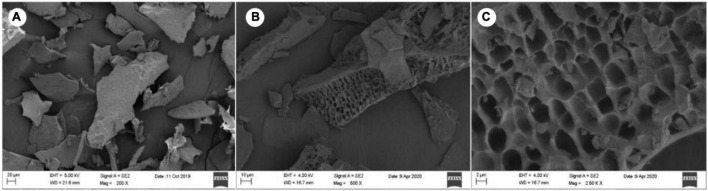
Scanning electron microscope (SEM) images of LJPS. **(A)** An overview of the uniform and flat surface of LJPS powder, image magnified 200 times; **(B)** image magnified 500 times. **(C)** The porous or honeycombed inside microstructure of LJPS, image magnified 250,000 times.

**FIGURE 2:**
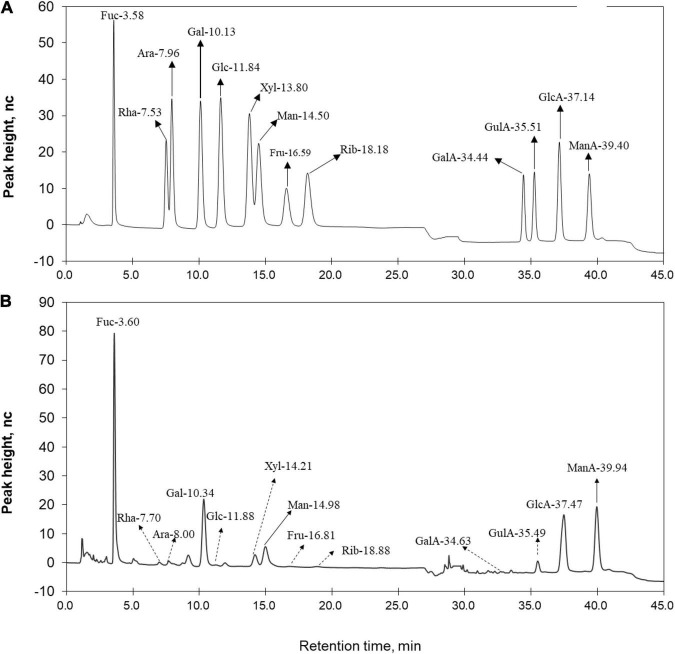
Ion chromatogram of **(A)** standard monomers and **(B)** LJPS. Each absorption peak was annotated based on the monomer name and retention time. Monomers with a molar proportion less than 5% were annotated with dotted lines.

The isolated LJPS had good aggregation and precipitation properties, and the freeze-dried powder had a uniform texture and flat surface (Figure 1A). After breaking down the outside surface, the porous or honeycombed microstructures were observed by SEM inspection (Figures 1B,C). The PS isolated from LJ consisted of 87.71% total sugar, 1.54% protein, 0.016% total phenols, and 10.73% sulfate (Table 1). The analysis of IC showed that LJPS was composed of Fuc, Rha, Ara, Gal, Glc, Xyl, Man, Fru, Rib, GalA, GluA, GlcA, and ManA, with a molar ratio of 35.71:1.48:0.28:13.16:0.55:2.97:6.92:0.58:0.41:0.14:3.16:15.84:18.79 (Figure 2 and Table 1). Thereof, the accumulated molar proportions of Fuc, Gal, Man, GlcA, and ManA reached 93.59%. In contrast, the molar percentages of Ara, Glc, Rib, and GalA (denoted with dotted lines in Figure 2) were less than 3%, and those compositions might vary considerably in different determinations.

**TABLE 1:** The monosaccharide components of polysaccharides (PSs) fractionated from *Laminaria japonica* polysaccharides (LJPS).

Chemical component	Total sugar,%	Protein,%	Total phenols,%	Sulfate,%
	87.71	1.54	0.016	10.73

**Monosaccharide components**	**RT^a^, min**	**nc^b^**	**Peak area, nc × min**	**Molar ratio,%**

Fuc	3.59 ± 0.00	70.91 ± 3.46	13.36 ± 1.06	35.71 ± 0.28
Rha	7.70 ± 0.01	0.39 ± 0.00	1.443 ± 0.00	1.48 ± 0.03
Ara	8.00 ± 0.01	1.29 ± 0.28	0.46 ± 0.11	0.28 ± 0.02
Gal	10.34 ± 0.01	21.29 ± 3.34	7.11 ± 1.19	13.16 ± 0.29
Glc	11.95 ± 0.01	0.78 ± 0.16	0.31 ± 0.06	0.55 ± 0.03
Xyl	14.21 ± 0.02	3.99 ± 0.76	1.53 ± 0.29	2.97 ± 0.08
Man	14.98 ± 0.03	5.36 ± 1.02	2.29 ± 0.44	6.92 ± 0.19
Fru	16.81 ± 0.03	0.29 ± 0.02	0.15 ± 0.00	0.58 ± 0.09
Rib	18.88 ± 0.03	0.36 ± 0.06	0.22 ± 0.04	0.41 ± 0.03
GalA	34.63 ± 0.00	0.34 ± 0.11	0.07 ± 0.02	0.14 ± 0.00
GulA	35.49 ± 0.02	3.94 ± 0.01	1.02 ± 0.00	3.16 ± 0.04
GlcA	37.47 ± 0.01	19.72 ± 4.03	6.18 ± 1.26	15.84 ± 0.11
ManA	39.94 ± 0.01	21.79 ± 4.04	7.10 ± 1.36	18.79 ± 0.16

*^a^RT, retention time; ^b^nc, nano coulomb (the unit of quantity of electric charge); nc × time, the peak area calculated by integration.*

### Fourier Transforms Infrared Spectra of LJ-Derived PS

**FIGURE 3:**
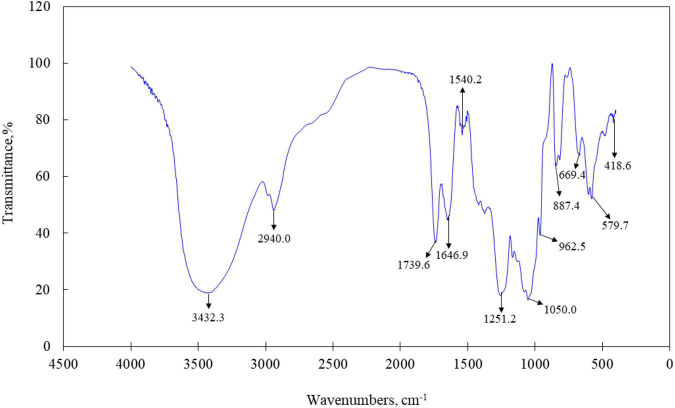
Fourier transforms infrared (FT-IR) spectra of LJPS.

The primary maxima of absorption bands in the IR spectra of LJPS are shown in Figure 3. FT-IR spectroscopy is an important analytical technique extensively used to study the molecular structures and conformations of macromolecules to identify the vibrations between different atoms in molecules. The transmittance spectrum with wavenumbers between 400 and 4,000 cm^–1^ was considered as a reflection of the structural characteristics of the LJPS, such as the glucosidic bonds and substituted groups (34, 35). As shown in Figure 3, the absorption band at 3,600–3,200 cm^–1^ was attributed to the stretching vibration of the –OH group, and the peaks in this region were indicative of the characteristic absorption for PS. In detail, the absorption peak presented at 3,432.3 cm^–1^ was identified as the O–H stretching vibration (O–H group), which was the typical peak of PS. The band at 2,940 cm^–1^ was C–H stretching vibration. The band at 1,646.9 cm^–1^ was ascribed to the C=O stretching vibration, which are indicative of carboxyl and carbonyl groups and characteristic of uronic acid (35, 36). The strong sharp absorption band at 1,251.2 cm^–1^ (S=O) stretching confirmed the existence of a significant amount of sulfate in the PSs, and the band at 1,050.0 cm^–1^ was attributed to the presence of the asymmetric O=S=O stretching vibration of a sulfate group. The sharp band at 887.76 cm^–1^ (C–S–O) suggested a pattern of sulfate substitution.

### Molecular Weight, Polydispersity, and Conformation Characteristics of LJ-Derived PS

**FIGURE 4:**
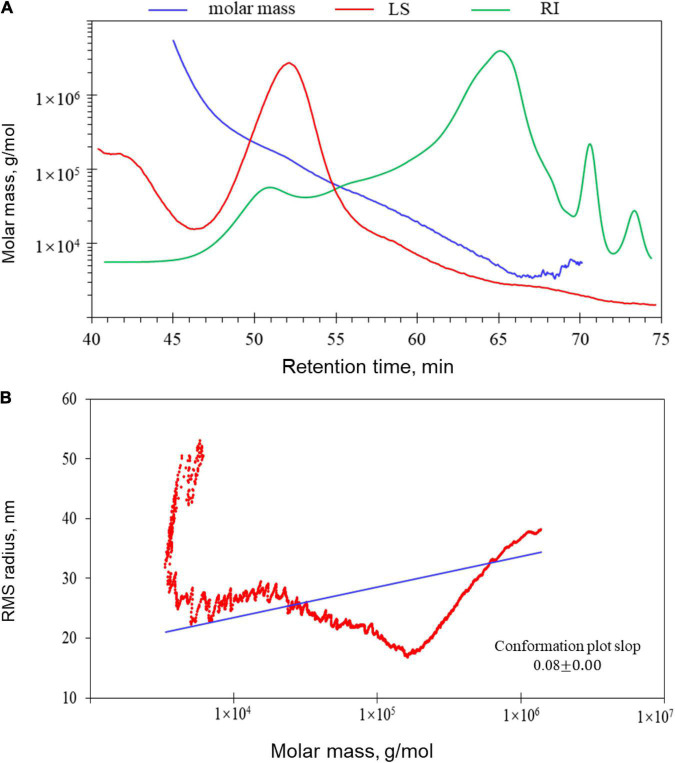
Gel permeation chromatography-refractive index-multiangle laser light scattering (GPC-RI-MALS) chromatograph with molecular weight distribution **(A)** and molecular conformation of LJPS **(B)**. **(A)** The variation tendencies of signals of the multi-angle laser scattering (LS), refractive index (RI), and fitted molar mass of LJPS. The red line indicates the variation tendency in the LS of LJPS with retention time, and the green line represents the trend in the RI of LJPS. The tendency of the red and green lines suggested the size of the PS molecules and their relative proportions contained in the tested sample. The blue line is the varying tendency of the molar mass fitted by the LS and RI signal of the PSs following the retention time. **(B)** Molecular conformation speculation plot, taking the log (molar mass) as the horizontal coordinate and log (RMS) as the vertical coordinate.

The molecular weights of LJPS are presented in Table 2. The Mw, Mn, and Mz of LJPS were calculated to be (1.17 ± 0.018) × 10^4^ (5.79 ± 0.006) × 10^4^, and (3.76 ± 0.012) × 10^5^ g/mol, respectively. The polydispersity coefficient of LJPS was 4.96 (Mw/Mn) or 32.11 (Mz/Mn), suggesting a relatively narrow range of molar mass distribution. In addition, the radius mean square (RMS) of LJPS was simultaneously analyzed as 26.1, 24.7, and 24.6 for the number-average radius (Rn), weight-average radius (Rw), and *Z*-average radius (Rz), respectively. The molecular weight characteristics of LJPS were further elucidated by the variation trend curve of molar mass (Figure 4), which displayed a rapidly decreasing laser scattering (LS) curve (red line) and gradually increasing RI line (green line), indicating that the LJPS contained relatively few proportional macromolecule PSs but had a high proportion of small molecules (the molar mass had polydispersity). The molar mass line (blue line) presented a rapid initial decrease and maintained a mild decline with an extension of the retention time, revealing a few macromolecular structures and more molecules with an intermediate molar mass in this polymer. The intersection of LS and RI curves corresponded to the value of the molar mass line of ∼10^4^, which revealed the distribution range of the molar mass of most PS molecules.

**TABLE 2:** The molecular weight, polydispersity, and root mean square (RMS) radius of LJPS.

Item*^a^*		Values
Molar mass (g/mol)	Mn	(1.17 ± 0.018) × 10^4^
	Mw	(5.79 ± 0.006) × 10^4^
	Mz	(3.76 ± 0.012) × 10^5^
Polydispersity	Mw/Mn	4.96 ± 0.019
	Mz/Mn	32.11 ± 0.037
Root mean square radius, RMS (nm)	Rn (nm)	26.1 ± 0.083
	Rw (nm)	24.7 ± 0.062
	Rz (nm)	24.6 ± 0.032

*^a^Mn, the number-average molecular weight; Mw, the weight-average molecular weight; Mz, the Z-average molecular weight; Rn, the number-average radius; Rw, weight-average radius; and Rz, Z-average radius.*

**FIGURE 5:**
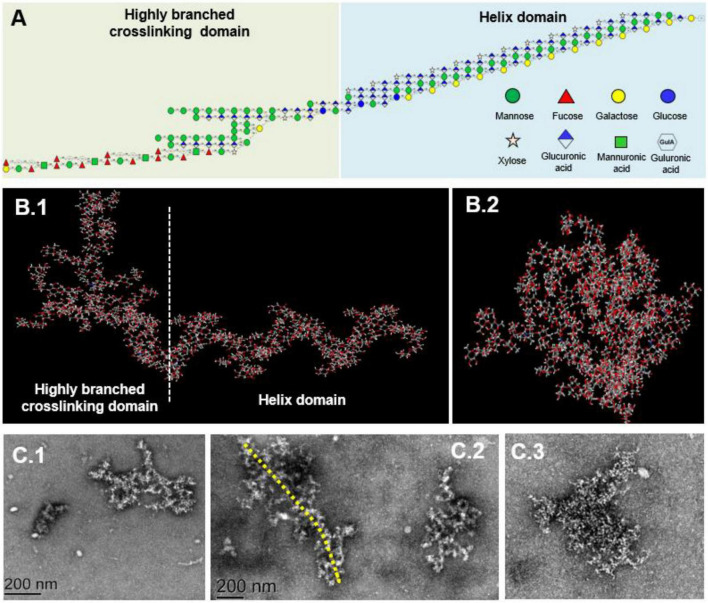
The proposed repeating units and molecular conformation of LJPS. **(A)** Structural formula, linkage of monomers, and proposed repeating units of LJPS. **(B1,B2)** Speculated 3-D molecular structure of LJPS from the side **(B1)** or front **(B2)** images by the Creators of GLYCAM-Web (http://glycam.org/). **(C1–C3)** Scanning images of LJPS dissolved in sodium dodecyl sulfate (SDS) and maintained with a dispersed status *via* transmission electron microscopy (TEM).

When the RMS value is more than 10 nm, taking the log(molar mass) as the horizontal coordinate and log(RMS) as the vertical coordinate to fit the conformation plot, the slope values suggested a biopolymer molecular configuration (37). As shown in Figure 4, the plot of RMS to the molar mass presented as a U-shaped structure, and the slope was 0.08 (Figure 4B), indicating that the spatial conformation of PS molecules comprised a dense crosslinking macromolecule with a highly branched structure. Correspondingly, TEM inspection verified that the unfolded LJPS molecules had a crosslinking network and/or long-chain status in SDS solution (Figure 5C). The subsequent spatial 3-D conformation speculation based on the structural parameters in this study confirmed the dense interconnected molecules with a highly branched structure in LJPS (Figure 5B).

### Glycosidic Linkages Between Monosaccharides of LJ-Derived PS

The GC-MS analysis revealed that LJPS was predominantly comprised of 21 monomer residues linked by different glycosidic bonds, such as non-reducing terminals of t-Rha*p*, t-Fuc*p*, t-Rib*p*, t-Xyl*p*, t-Man*p*A, t-Glc*p*A, and t-Galp with the molar ratios of 0.47, 3.74, 0.43, 4.25, 3.44, 1.29, and 0.51%, respectively (Table 3). Other monomers compositions, such as 1,3-Fucp; 1,3-Fucp; 1,2-Xyl*p*; 1, 3-Glc*p*A; 1, 2-Man*p*; 1, 4-Man*p*A; 1, 4-Gal*p*; 1, 4-Glc*p*A; 1, 2, 3-ManpA; 1,3,4-GlcpA; 1, 4-GulpA; 1,2,3,4-Galp; 1,2,6-GlcpA; and 1, 2, 3, 6-ManpA with the molar ratios of 1.96, 1.86, 0.99, 4.46, 10.50, 19.07, 13.81, 18.36, 1.93, 5.89, 3.99, 0.54, 1.55, and 0.94%, respectively, were identified as the intrachain residues. The main branching points were at 1,2,3-ManpA; 1,3,4-GlcpA; 1,2,3,4-Galp; 1,2,6-GlcpA; and 1,2,3,6-ManpA comprising 1.93, 5.89, 0.54, 1.55, and 0.94%, respectively. Of those glycosidic residues, irrespective of the glycosidic bond types, seven monomer residues had relative high proportions (> 3%), namely, Fuc (7.567%), Xyl (5.25%), ManA (25.38%), GlcA (31.56%), Gal (14.85%), Man (10.51%), and GulA (3.99%). Those might be the main constituents of LJPS molecules. In addition, the degree of branching (DB) value was 24.99% for LJPS, which was the proportion of the accumulated numbers of terminal and branch residues accounting for the total amount of saccharide residues. This revealed that the LJPS was a highly branched molecule.

**TABLE 3:** Glycosidic linkages among the monomer residues and molar proportions of LJPS.

Glycosidic linkages	Derivative name	RT^a^	mol,%
t-Rha(p)	1,5-di-*O*-acetyl-6-deoxy-2,3,4-tri-*O*-methyl rhamnitol	6.32	0.47
t-Fuc(p)	1,5-di-*O*-acetyl-6-deoxy-2,3,4-tri-*O*-methyl fucitol	7.67	3.74
t-Rib(p)	1,5-Di-O-acetyl-1-deuterio-2,3,4-tri-O-methyl-D-ribitol	7.98	0.43
t-Xyl(p)	1,5-di-*O*-acetyl-2,3,4-tri-*O*-methyl xylitol	7.98	4.25
t-Man(p) A	1,5-di-*O*-acetyl-2,3,4,6-tetra-*O*-methyl mannitol	9.62	3.45
t-Glc(p)A	1,5-di-*O*-acetyl-2,3,4,6-tetra-*O*-methyl glucitol	9.72	1.3
1, 3-Fuc(p)	1,3,5-tri-*O*-acetyl-6-deoxy-2,4-di-*O*-methyl fucitol	10.39	1.96
t-Gal(p)	1,5-di-*O*-acetyl-2,3,4,6-tetra-*O*-methyl galactitol	10.77	0.5
1, 2-Fuc(p)	1,2,5-tri-*O*-acetyl-6-deoxy-3,4-di-*O*-methyl fucitol	11.2	1.86
1, 2-Xyl(p)	1,2,5-tri-*O*-acetyl-3,4-di-*O*-methyl xylitol	12.29	0.99
1, 3-Glc(p)A	1,3,5-tri-*O*-acetyl-2,4,6-tri-*O*-methyl glucitol	13.08	4.46
1, 2-Man(p)	1,2,5-tri-*O*-acetyl-3,4,6-tri-*O*-methyl mannitol	13.31	10.5
1, 4-Man(p)A	1,4,5-tri-*O*-acetyl-2,3,6-tri-*O*-methyl mannitol	13.87	19.07
1, 4-Gal(p)	1,4,5-tri-*O*-acetyl-2,3,6-tri-*O*-methyl galactitol	14.58	13.81
1, 4-Glc(p)A	1,4,5-tri-*O*-acetyl-2,3,6-tri-*O*-methyl glucitol	15.00	18.36
1, 2, 3-Man(p)A	1,2,3,5-tetra-*O*-acetyl-4,6-di-*O*-methyl mannitol	16.04	1.93
1, 3,4-Glc(p)A	1,3,4,5-tetra-*O*-acetyl-2,6-di-*O*-methyl glucitol	17.21	5.89
1, 4-Gul(p)	1,4,5-tetra-*O*-acetyl-2,3,6-di-*O*-methyl guluronate	17.64	3.99
1, 2, 3, 4-Gal(p)	1,2,3,4,5-penta-*O*-acetyl-6-*O*-methyl galactitol	17.92	0.54
1, 2, 6-Glc(p)A	1,2,5,6-tetra-*O*-acetyl-3,4-di-*O*-methyl glucitol	19.22	1.55
1, 2, 3, 6-Man(p)A	1,2,3,5,6-penta-*O*-acetyl-4-*O*-methyl mannitol	22.39	0.94

*^a^RT, retention time.*

### Nuclear Magnetic Resonance Spectrum of LJ-Derived PS

**FIGURE 6:**
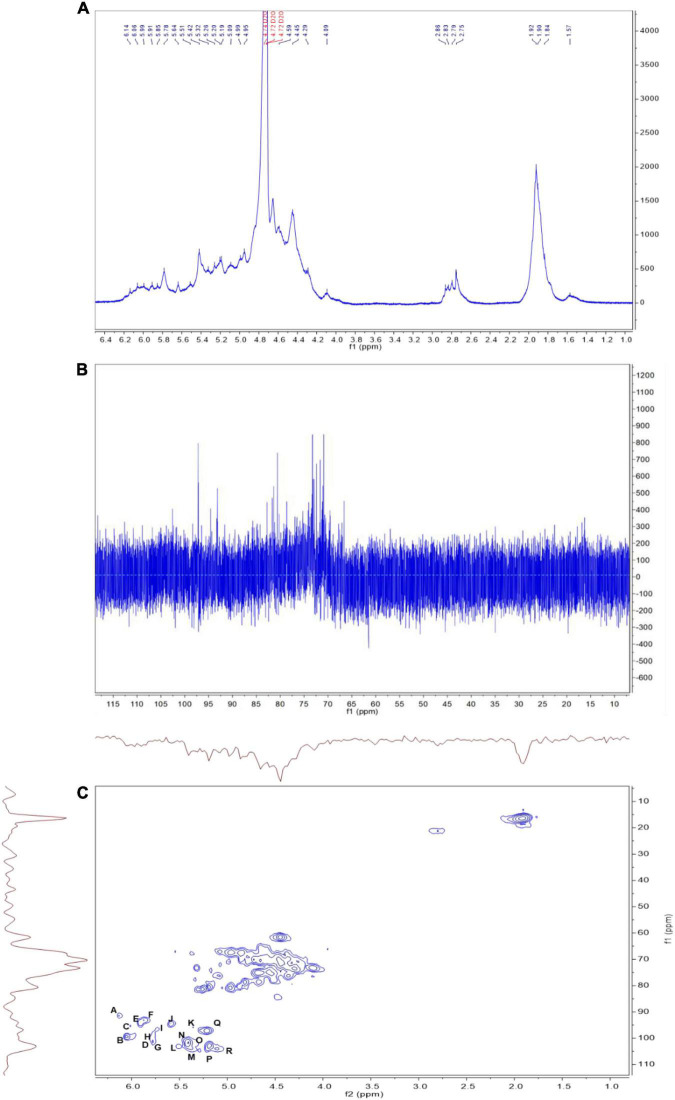
The ^1^H **(A)**, ^13^C **(B)**, and heteronuclear singular quantum correlation (HSQC) **(C)** nuclear magnetic resonance (NMR) spectrum of LJPS in D_2_O solution at 25^°^C. A, B, C…R in **(C)** indicate the correlation between carbon 1 (C1) and hydrogen 1 (H1) of residue A. The order of residues A, B, C…R is consistent with that in Table 4.

The linkages between glycosyl residues in LJPS were further investigated by NMR analyses, such as ^1^H NMR, ^13^C NMR, and HSQC spectra (Figure 6), with the assistance COSY, HMBC, and NOESY (not shown). The ^1^H NMR spectrum signals were mainly distributed in the 1.5–6.3 ppm range, the chemical shifts between δ 3.2 and 4.0 ppm were the protons of the glycosidic ring, and δ 4.09, 4.45, 4.65, 4.95, 5.42, 5.64, and 5.78 were assigned to terminal protons, whereas other signals peaks (chemical shift) were distributed in δ 2.6–3.0 and 4.3–5.5 ppm. Analysis of the ^13^C NMR spectrum revealed that the carbon signals were primarily distributed at chemical shift δ –120 ppm. The anomeric carbons were at δ 91.48, 93.49, 94.66, 97.31, 99.52, 101.1, 101.83, and 103.21 ppm, and the signals of anomeric carbons were mainly found at δ 91–105 ppm. However, the signal peaks of the chemical shift were at δ 84.61, 81.96, 81.02, 80.59, 79.55, 79.01, 78.71, 77.54, 76.70, 73.31, 71.20, 67.69, 65.80, and 62.02, which scattered in the δ 60–105 ppm range. In addition, regarding the correlations of C/H in the heteronuclear singular quantum correlation (HSQC) spectrum (Figure 6C), 18 cross signals, with the ^1^H being from 4.6 to 6.5 ppm and ^13^C in the range of 90–115 ppm, further demonstrated the presence of A-R residues, which are represented in Figure 6 and summarized in Table 4. Combining the information of HMBC and NOESY analysis, the structure of this PS could be delineated as follows: the backbone of LJPS was composed of →2)-α-D-Manp-(1 → 3)-α-D-Fuc*p*-(1 → 3)-α-D-Man*p*A-(1 → 2)-α-D-Fuc*p*-(1 → 2)-α-D-Man*p*-(1 → 2)-α-D-Xylp-(1 → 6)-α-D-Manp-(1 → 3)-α-D-Gal*p*-(1 → 6)-α-D-Man*p*-(1 → 2)-α-D-Xyl*p*-(1 → 4)-α-D-Glc*p*-(1 → 6)-α-D-Glc*p*-(1 → 4)-α-D-Gal*p*-(1 → 4)-α-D-Glc*p*-(1 → 4)-α-D-Gal*p*-(1 →; and the branch chain included: →4)-α-D-Gulp-(1 → was linked to the backbone chain by O-2 of 2, 3)-α-D-Man*p*A; →3)-α-D-GlcpA-(1 → was connected with main chain by O-2 of 2, 6)-α-D-Glc*p*A; →4)-α-D-GlcpA-(1 → 4)-α-D-GlcpA-(1 → 4)-α-D-Man*p*A-(1 → 4)-α-D-ManpA-(1 → linked to the main chain by O-3 of 3,4) –α-D-GlcpA; →4)-α-D-ManpA-(1 → was linked to the backbone by O-2 of 2, 3, 6)-α-D-ManpA; →4)-α-D-GlcpA-(1 → was connected with the main chain by O-3 of 2, 3, 6)-α-D-ManpA; →2)-α-D-Manp-(1 → was linked with backbone by O-2 of 2,3,4)-α-D-Galp; and →2)-α-D-Manp-(1 → was linked to the main chain by O-4 of 2,3,4)-α-D-Gal*p*. Therefore, based on the results of glycosidic linkage and NMR spectra analyses, the possible monomers linkage of LJPS is shown Figure 5A.

**TABLE 4:** The ^1^H and ^13^C nuclear magnetic resonance (NMR) chemical shifts of LJPS recorded in D_2_O at 25°C.

Number	Glycosyl residues	Chemical shift (ppm), δ						
		C1/H1	C2/H2	C3/H3	C4/H4	C5/H5	C6/H6	H6b
A	→1)-α-D-ManpA-(2,3,6→	91.58	72.31	82.5	70.82	75.81	71.76	
		6.13	4.57	4.63	5.16	4.84	4.96	4.86
B	→1)-α-D-Xylp-(2→	99.26	73.16	74.93	75.96	70.87		
		6.06	4.44	4.58	4.86	4.88		
C	→1)-α-D-Fucp	95.86	75.17	72.58	78.8	77.69	62.67	
		6.01	4.28	4.43	4.64	4.47	4.95	
D	→1)-α-D-Galp-(2,3,4→	99.66	72.42	84.1	70.96	75.14	63.45	
		5.98	4.7	4.8	4.86	4.57	4.63	4.74
E	→1)-α-D-Fucp-(3→	94.87	83.62	78.97	86.22	63.64	82.15	
		5.9	5.13	4.87	5.06	4.76	4.64	4.79
F	→1)-α-D-Fucp-(2→	93.47	72.5	71.05	72.71	77.4	63.45	
		5.86	4.7	4.9	4.75	4.63	4.74	4.62
G	→1)-α-D-Gulp-(4→	101.27	72.75	73.34	78.69	77.11	63.14	
		5.77	4.97	4.75	4.73	4.46	4.86	4.64
H	→1)-α-D-GlcpA- (2,6→	99.66	72.13	73.34	78.77	76.29	63.04	
		5.78	5.03	4.63	4.53	4.5	4.76	4.24
I	→1)-α-D-ManpA- (2,3→	96.53	75.34	78.24	80.84	76.28	62.92	
		5.73	4.22	4.4	4.59	4.59	4.79	4.97
J	→1)-α-D-Manp- (2→	95.02	73.37	73.58	80.84	72.34	62.59	
		5.59	4.48	4.72	4.59	4.72	4.81	4.65
K	→1)-α-D-ManpA	95.86	73.73	75.63	71.87	72.47	67.97	
		5.36	4.5	4.65	4.45	4.84	4.9	
L	→1)-α-D-Xylp	103.41	76.07	75.03	78.56	68.81		
		5.51	4.19	4.56	4.34	4.47		
M	→1)-α-D-GlcpA- (3→	104.4	73.32	73.57	80.83	72.19	62.45	
		5.37	4.48	4.74	4.58	4.84	4.77	4.72
N	→1)-β-D-GlcpA- (4→	102.26	75.06	74.02	77.69	67.96	62.14	
		5.41	4.2	4.56	4.33	4.48	4.63	4.84
O	→1)-α-D-GlcpA	101.79	71.5	70.05	71.71	76.4	62.45	
		5.34	4.7	4.9	4.75	4.63	4.74	4.62
P	→1)-α-D-Galp- (4→	103.25	73.44	73.58	80.7	72.41	62.66	
		5.18	4.49	4.73	4.61	4.85	4.82	4.67
Q	→1)-α-D-ManpA- (4→	97.31	71.78	72.34	77.69	75.88	62.14	
		5.22	4.96	4.75	4.73	4.49	4.85	4.64
R	→1)-α-D-GlcpA- (3,4→	104.08	78.35	77.61	63.14	75.6	71.5	
		5.08	4.59	5.12	4.63	4.84	4.95	4.83

### Three-Dimensional Structure and Spatial Conformation of Long-Chain LJ-Derived PS

Combining all structural parameters involving the monosaccharide composition, glycosidic type, sequences, and linking position of branched chains to fit the potential molecular structure and morphology, the structural formula indicated that the LJPS molecule was a long-chain PS with 176 monomers and highly branched structural characteristics (Figure 5A and Supplementary Figure 1). The spatial conformation results showed that there were 32 potential spatial conformations with different folding angles and torsions (Supplementary Figure 2). Additionally, the 3-D spatial conformation video showed that the long-chain LJPS isolated in this study was a denser interconnected macromolecule with highly branched and helix domains (Figure 5B and Supplementary Video 1). The profile from the side (Figure 5B1) and front (Figure 5B2) were well matched with the scanning images of TEM (Figure 5C), showing the overall shape and spatial conformation of the long-chain LJPS. The structural characteristics and chain spatial conformation confer the bioactivities of PSs (38). However, owing to the complex compositions, highly branched structure, large molecular weight, viscosity, and aggregating properties in solution, it was difficult to characterize the spatial conformation of PS molecules. In this study, we preliminarily explored the spatial structure of long-chain LJPS; however, the relationship between this unique molecular conformation and its biological activity needs to be further studied.

### Cellular Toxicity and Anti-inflammatory Effect of LJ-Derived PS on Mouse Macrophage Cells

**FIGURE 7:**
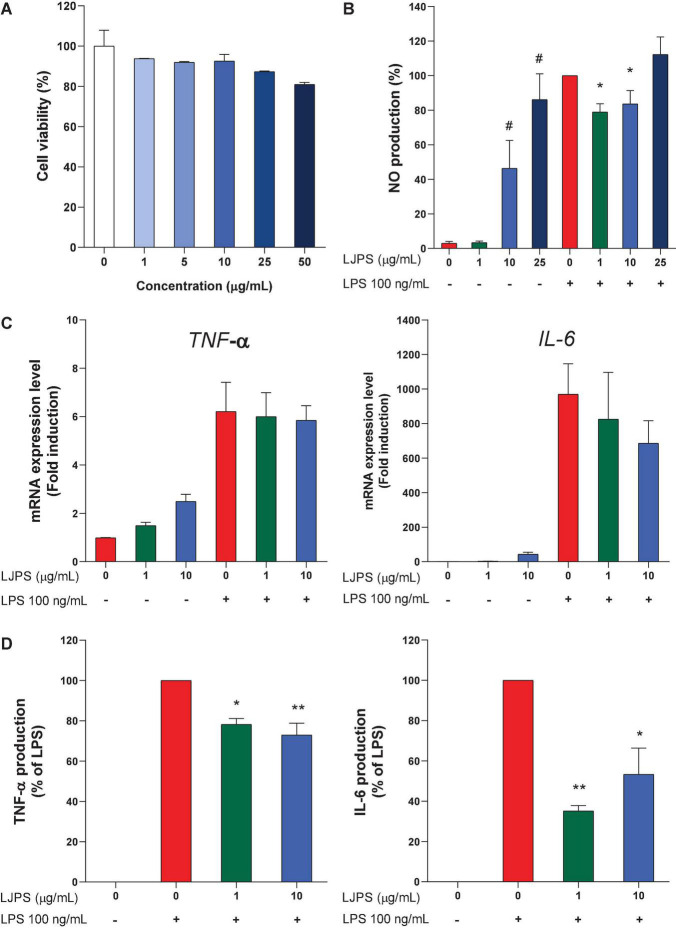
Cellular toxicity and immunomodulating effects of LJPS in mouse macrophage RAW 264.7 cells. **(A)** The effect of LJPS on the viability of RAW 264.7 cells. RAW cells were treated with various concentrations of LJPS (0–50 μg/ml) for 24 h. **(B)** The effects of LJPS on NO production with and without LPS stimulus in RAW 264.7 cells. RAW cells were starved overnight and pretreated with LJPS (1, 10, or 25 μg/ml) for 6 h and then stimulated with LPS (100 ng/ml). Values are the mean ± SEM. # and * indicate a significant difference compared with that in the control without and with LPS stimulus, respectively (*p* < 0.05). **(C,D)** RAW cells were starved overnight and pretreated with LJPS (1 or 10 μg/ml) for 6 h and then stimulated with LPS (100 ng/ml). At the end of treatment, RNA and supernatants were collected to detect mRNA and protein expression of IL-6 and TNF-α. Values are the mean ± SEM of three independent experiments. * Indicates a significant difference (*p < 0.05, **p < 0.01). LJPS, *Laminaria japonica* derived polysaccharide; LPS, lipopolysaccharide; NO, nitric oxide; TNF-α, tumor necrosis factor-α; IL-6, interleukin-6.

Various concentrations of LJPS were administered to the mouse macrophage RAW 264.7 cells for 24 h to determine the potential cytotoxicity of LJPS. As shown in Figure 7A, no significant cytotoxicity was observed after treatment for 24 h, although the highest concentration of LJPS, 50 μg/ml, seemed to result in a slight reduction in cell viability (*p* = 0.069). Therefore, further experiments with RAW 264.7 cells were performed with < 10 μg/ml LJPS. Next, we investigated how LJPS regulates the activation of macrophages with and without LPS endotoxin stimulation by measuring NO production, as well as inflammatory cytokines, such as IL-6 and TNF-α. As shown in Figure 7B, LJPS at both 10 and 25 μg/ml significantly induced NO production without the LPS stimulus, whereas LPS-induced NO production was significantly decreased by LJPS treatment at both 1 and 10 μg/ml concentrations in mouse macrophage cells (Figure 7B). However, 25 μg/ml LJPS resulted in a significant increase in NO production regardless of LPS challenge. This indicated the biphasic regulatory property of LJPS in anti-inflammation and immunomodulation only within a certain range of concentrations (≤ 10 μg/ml). LJPS treatment did not significantly change the mRNA expression of either *Il-6* or *Tnf-*α regardless of LPS stimulation in RAW 264.7 cells (Figure 7C). Unlike the biphasic regulatory effect of LJPS on NO production, LJPS itself did not induce the production of the proinflammatory cytokines TNF-α and IL-6 in RAW 264.7 cells. However, LJPS significantly attenuated LPS-induced TNF-α and IL-6 production, compared with that in the LPS only treated group (Figure 7D), suggesting an anti-inflammatory capacity and its translational regulation.

## Discussion

In the present study, a novel long-chain PS was isolated from LJ *via* water extraction and alcohol precipitation methods and purified by semipermeable membrane dialysis (molar mass > 3,500 D, DP > 20). LJPS powder presented with a flat appearance and honeycomb or porous microstructure by SEM inspection, which was consistent with the work of Sun et al. (39), who reported that the PS from LJ, *via* water extraction, appeared to have a flat smooth surface. However, Rodriguez-Jasso et al. (40) demonstrated that the PS from LJ isolated by acid, alkaline, or microwave-assisted extraction appeared to have a rough surface. The different surface topographies of the PSs are probably ascribed to the different extraction procedure (31).

The analysis of IC showed that LJPS was composed of Fuc, Rha, Ara, Gal, Glc, Xyl, Man, Fru, Rib, GalA, GluA, GlcA, and ManA, with a molar ratio of 35.71:1.48:0.28:13.16:0.55:2.97:6.92:0.58:0.41:0.14:3.16:15.84:18.79. Similarly, Gao et al. (24) found that the PS extraction from LJ *via* an acid-assisted method consisted of Fuc, Gal, ManA, GlcA, Man, Xyl, and Ara, with a molar ratio of 44.16:3.86:26.19:15.53:6.53:62.11,19.56,12.92. Additionally, Imbs et al. (41) observed that the PS from brown algae was composed of Fuc, Gal, Man, Xyl, Glc, ManA, and GlcA with a molar ratio of 45.0:18.0:16.2:6.0:8.8:6.0. In those studies, LJPS was obtained by water extraction and ethanol precipitation procedures, and similar constituents were identified. Fuc, Gal, Man, ManA, and GlcA were the predominant constituents with the accumulated proportions of more than 80%, which suggested that the five monomers might be the predominant and species-specific intrachain monosaccharide components of LJPS. However, discrepant findings were also reported, for example, Zha et al. (28) showed that LJPS merely contained monosaccharides of Gal (50.60%), Man (17.33%), and Glc (32.06%), with no uronic acids. Similarly, Peng et al. (42) reported that the PS fractioned from LJ was mainly composed of Ara, Xyl, Man, Glc, and Gal in a molar ratio of 1:0.17:1.54:2.64:0.18. Thus, the discrepant monomer compositions of LJPS in various studies could be mainly attributed to the different LJ source (i.e., distinct growth environment and harvesting period) and the numbers of monomer standard substances that were applied in the process of composition determination. Of note, if the standard substances did not include uronic acids, uronic acids contained in PS would not be identified, which will lead to human error. The analysis of FT-IR spectroscopy revealed the high sulfate radicals content, which was consistent with the determination of sulfate content. A sulfate group is a characteristic component of PSs from brown seaweeds (43, 44). Similarly, Gao et al. (24) found that LJPS with a greater molar mass and complex crosslinking structure had more sulfate groups and higher bile acid-binding capacity.

The analysis of glycosidic linkages indicated that seven main monomers residues had relative high proportions (> 3%), namely, Fuc (7.567%), Xyl (5.25%), ManA (25.38%), GlcA (31.56%), Gal (14.85%), Man (10.51%), and GulA (3.99%), and the intrachain glycosidic linkages included (1 → 3), (1 → 2), (1 → 6), and (1 → 3, 4) bonds. Similarly, Gao et al. (24) observed the dominant residues of GlcA, ManA, Man, and Fuc and glycosidic linkages of (1 → 3), (1 → 2), (1 → 3, 4) in PS isolated from LJ. The ratio between the terminal units and the branching points was 1.3, suggesting that the number of the terminal units was a slightly higher than the number of the branching points in PS molecules.

In addition, the DB value was 24.99% for LJPS, which was the proportion of the accumulated numbers of terminal and branch residues accounting for the total amount of saccharide residues. This revealed that LJPS is a highly branched molecule (24, 45). Additionally, these glycosidic binding types between the adjacent monomers of PS are a vital influencing factor on the antioxidant capacity of PS (3, 39). Previous studies documented that phytogenic PS with a backbone containing 1,3- or 1,6-glycosidic linkages in the backbone has stronger hydroxyl radical scavenging activity (46). Thus, the monomer residues and glycosidic bonds contributed to the bioactivities of LJPS.

For RMS values more than 10 nm, taking the log (molar mass) as the horizontal coordinate and the log (RMS) as the vertical coordinate to fit the conformation plot, the slope values suggested the biopolymer molecular configuration (37). In this study, the plot of RMS to molar mass presented with a U-shaped structure, and the slope was 0.08, indicating that the conformation of the PS molecule was a highly branched molecule. In addition, TEM image inspection showed the crosslinking network structure of the unfolded LJPS molecules. The average molar mass and polydispersity are the pivotal parameters of molecular characteristics, as they play a pivotal role in the dynamic properties and bioactivities (3, 14). In this study, the Mw, Mn, and Mz of LJPS were determined to be (1.17 ± 0.018) × 10^4^, (5.79 ± 0.006) × 10^4^, and (3.76 ± 0.012) × 10^5^ g/mol, respectively. This was consistent with the results of Zha et al. (47), who observed that the Mw of LJPS was approximately 1.9–4.3 × 10^4^ D. Similarly, Peng et al. (42) showed that the molecular weight of one type of PS extracted from *L. japonica* was approximately 2.31 × 10^4^ D. The average molar mass and polydispersity are the pivotal parameters of molecular characteristics, which determine dynamic properties and bioactivities (11, 14). The NMR analysis further provided structural information about the monosaccharide composition, residue sequence, linkage types, and conformation of glycosyl-residues (43, 48).

Combining all structural characteristic parameters to fit the potential spatial configuration of LJPS revealed that the long-chain LJPS is a dense complex macromolecule with highly branched and helix domains, with a porous internal construction. The mean Mw was (5.79 ± 0.06) × 10^4^, and the polydispersity was 4.96. The main compositions consisted of Fuc, Gal, Man, ManA, and GlcA, and the primary intrachain glycosidic bonds included (1 → 3), (1 → 2), (1 → 4), (1 → 3, 4), and (1 → 2, 4).

In this study, another interesting finding was that LJPS had a biphasic regulatory effect on RAW 264.7 cells, in that LJPS at 10 and 25 μg/ml significantly induced NO production without LPS stimulus, whereas LPS-induced NO production was significantly decreased by LJPS treatment at both 1 and 10 μg/ml concentrations in mouse macrophage cells. Similarly, we observed that LJPS tended to augment the mRNA expression of *Tnf-*α and *Il-6* without external stimuli (expression at a low level) but lowered the mRNA expression of *Tnf-*α and *Il-6* in LPS-challenged RAW 264.7 cells. Furthermore, the production of *TNF-*α and *IL-6* in LPS-induced RAW 264.7 cells was significantly decreased with LJPS supplementation at both 1 and 10 μg/ml concentrations, suggesting its anti-inflammatory capacity and its translational regulation. Thus, our findings revealed the biphasic regulatory properties of LJPS with respect to anti-inflammation and immunomodulation. This was consistent with the results of Zha et al. (47), who reported that LJPS had no cell toxicity to RAW 264.7 cells and that LJPS inhibited ox-LDL-induced foam cell formation and intracellular lipid accumulation in RAW 264.7 cells. Similarly, in a parallel study, Fang et al. (49) documented the immunomodulatory activity of LJPS with macrophages, showing the enhanced production of NO, TNF-α, IL-1β, IL-6, and IL-10 *via* associations with the toll like receptor (TLR) 4 receptor and the activation of mitogen-activated protein kinase (MAPK) and nuclear factor κB (NF-κB) signaling pathways. Similarly, certain phytogenic PSs generally have multiple biological activities (14). In addition, Wang et al. (50) observed the bidirectional immunomodulatory activities of purified PSs from *Pleurotus nebrodensis*. The PSs isolated from alfalfa were also found to have biphasic regulatory effects on immuno-stimulation (51). The bioactivities of plant-derived PS are tightly linked with its molecular structure, mainly with respect to the monomer constituents, molar mass, molecular configuration, and glycosidic linkages, and especially the unique spatial conformation (14, 31). Furthermore, the mechanism underlying the biphasic regulatory effects of plant-derived PSs was considered to be associated with the activation and/or overactivation of relative signaling pathways (50, 51).

The structural characteristics and chain spatial conformation are responsible for the bioactivities of PSs (3, 38). However, owing to its complex composition, highly branched structure, large molecular weight, viscosity, and aggregating properties in solution, it was difficult to characterize the spatial conformation of PS molecules. In this study, we preliminarily explored the spatial structure of long-chain LJPS, revealing that the LJPS was a long-chain and denser crosslinking macromolecule with highly branched and helix domains in the terms of its spatial conformation; it also had biphasic immunomodulation and anti-inflammatory bioactivities. However, further studies are needed to investigate the interrelation between the structural characteristics and their bioactivities.

## Conclusion

In summary, a long-chain sulfated polysaccharide with an Mw of 5.79 × 10^4^ g/mol was obtained from LJ by water extraction-ethanol precipitation-dialysis methods. The long-chain LJPS was composed of Fuc, Rha, Ara, Gal, Glc, Xyl, Man, Fru, Rib, GalA, GluA, GlcA, and ManA, with a molar ratio of 35.71:1.48:0.28:13.16:0.55:2.97:6.92:0.58:0.41:0.14:3.16:15.84:18.79. Thereof, Fuc, Gal, Man, GlcA, and ManA were the predominant components with an accumulated proportion of 93.6%. The molecules of long-chain LJPS were comprised of 21 glycosidic linkage types and seven main monomer residues, such as Fuc (7.567%), Xyl (5.25%), ManA (25.38%), GlcA (31.56%), Gal (14.85%), Man (10.51%), and GulA (3.99%). The structure and conformation analysis indicated that LJPS was a cross-linked network macromolecule with a porous or honeycomb microstructure. Regarding the molecular conformation, LJPS was a multi-branched long-chain macromolecule and appeared in a denser crosslinking network with highly branched and helix domains in the terms of morphology. Long-chain LJPS had no toxicity to RAW 264.7 cells and exhibited biphasic immuno-stimulatory or anti-inflammatory capacity. Thus, our findings revealed that the long-chain LJPS is a potential candidate immunopotentiating and anti-inflammatory functional food, and this study provides a feasible approach to decipher the structural characteristics and spatial conformation of plant-derived PSs.

## Data Availability Statement

The original contributions presented in the study are included in the article/Supplementary Material, further inquiries can be directed to the corresponding author/s.

## Author Contributions

JC and YW: investigation, formal analysis, and writing—original draft. EK and CZ: investigation and formal analysis. GZ and YL: conceptualization, supervision, funding acquisition, and writing—original draft, review, and editing. All authors contributed to the article and approved the submitted version.

## Conflict of Interest

The authors declare that the research was conducted in the absence of any commercial or financial relationships that could be construed as a potential conflict of interest.

## Publisher’s Note

All claims expressed in this article are solely those of the authors and do not necessarily represent those of their affiliated organizations, or those of the publisher, the editors and the reviewers. Any product that may be evaluated in this article, or claim that may be made by its manufacturer, is not guaranteed or endorsed by the publisher.

## Footnotes

1
http://glycam.org/
