# Supplementary material for: Structural Characteristics and Immunomodulatory Effects of a Long-Chain Polysaccharide From Laminaria japonica
Source: Front Nutr. 2022 Mar 28;9:762595. doi: 10.3389/fnut.2022.762595 (PMC8996131; doi:10.3389/fnut.2022.762595)
Supplement: Supplementary file 1 [file Data_Sheet_1.docx]

Supplementary Material

**Supplementary Figure 1.** The structural formula and linkage of monomers in LJPS molecule.


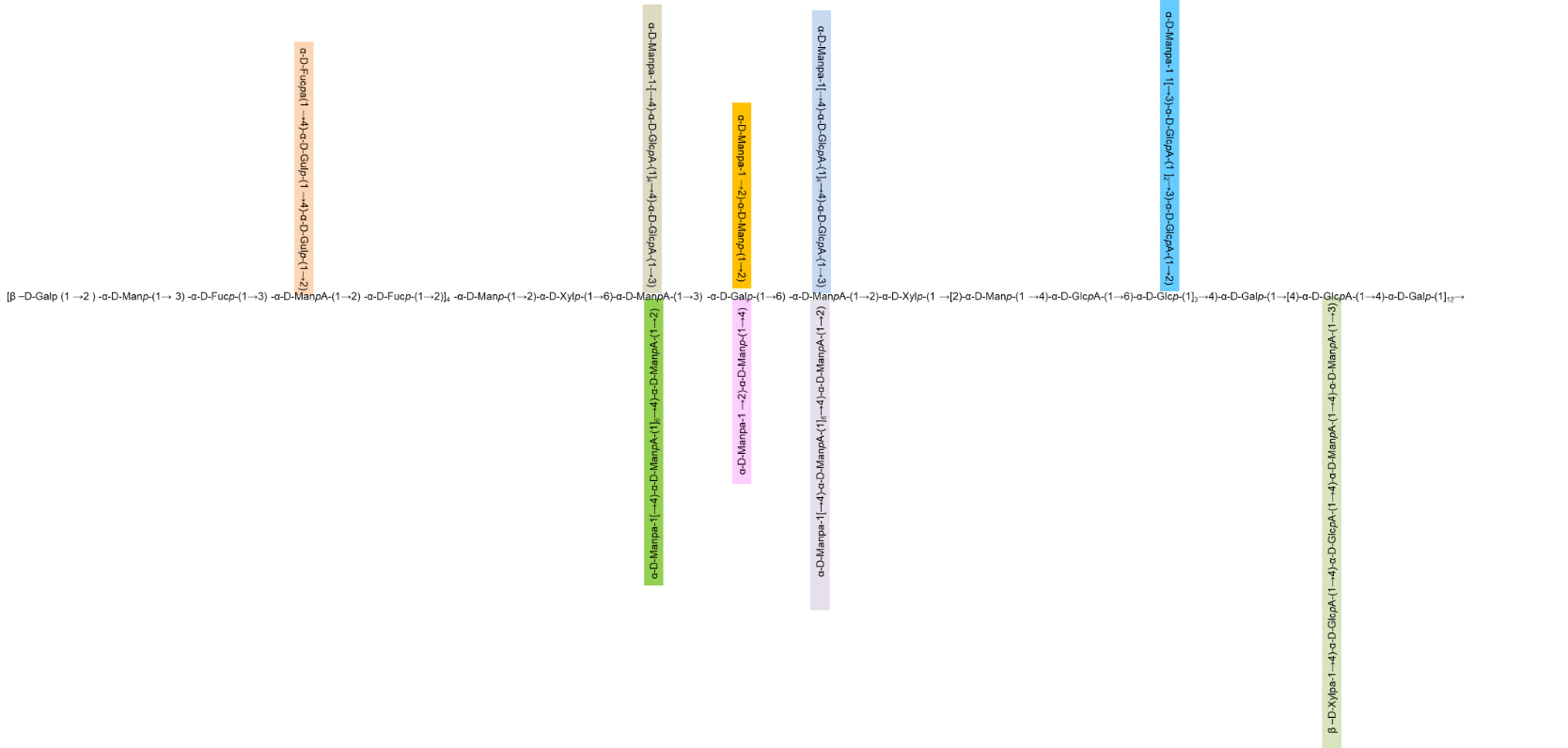


**Supplementary Figure 2. The 32-potential molecular conformation of LJPS with different folding angles**


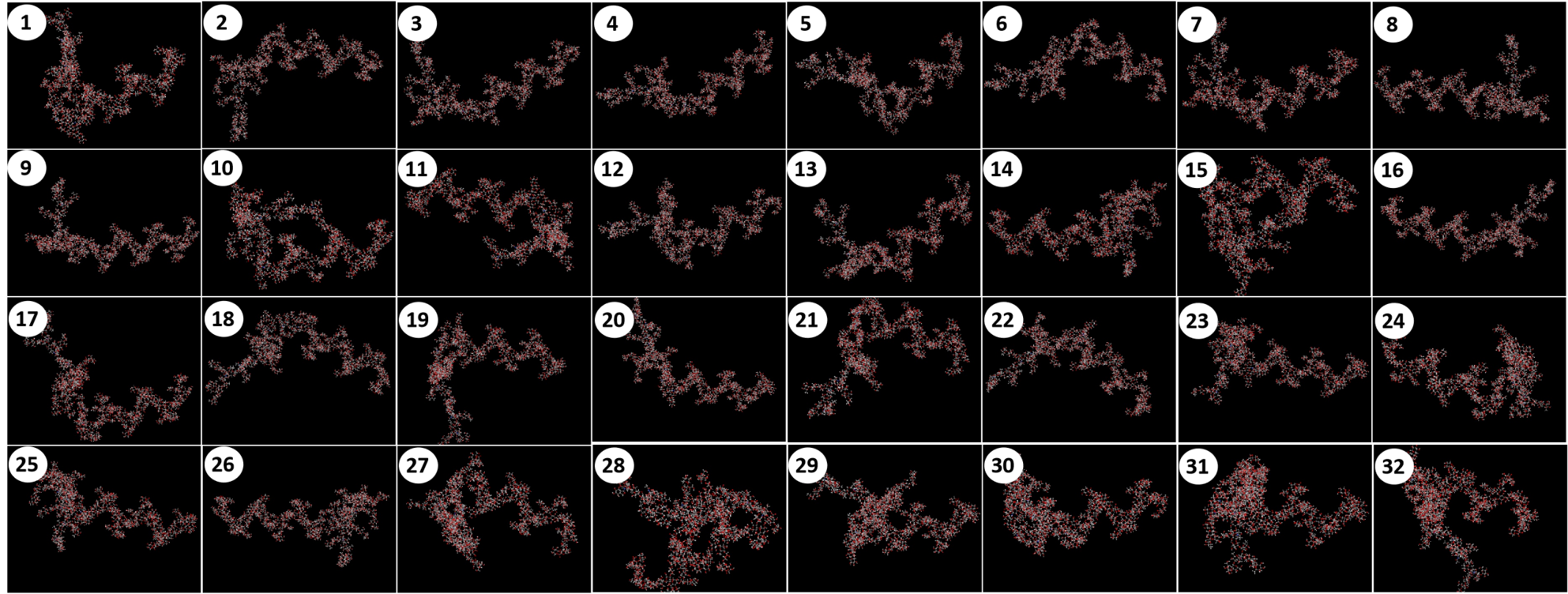


**Supplementary Video 1.**  The 3-d conformation of LJPS molecule.

The rotating 3 directional conformation molecule of LJPPS clearly shown the highly branched long-chain with branched and helix domain

*Please refer to the submitted video (mp4) file.*
